# Inter-brain synchrony between undergraduate students during a naturalistic online seminar predicted greater relational satisfaction and task performance

**DOI:** 10.3389/fnins.2025.1705767

**Published:** 2025-11-11

**Authors:** Atiqah Azhari, Ashvina Rai, Han Sheng Ho, Ajevan Jegathisan, Shafeeqah Gill, Noelle Norfor, Zan Chen

**Affiliations:** Psychology Programme, School of Humanities and Behavioural Sciences, Singapore University of Social Sciences, Singapore, Singapore

**Keywords:** online collaborative learning, virtual learning, inter-brain synchrony (IBS), fNIRS, hyperscanning, relational satisfaction, task performance, prefrontal cortex

## Abstract

As higher education increasingly transitions to online platforms such as Zoom, understanding the mechanisms that underlie effective virtual collaboration has become essential. Prior research has shown that relational quality, trust, and communication strongly influence online collaborative learning; however, the real-time cognitive and affective dynamics underpinning these interactions are yet to be elucidated. This study addresses this gap by examining inter-brain synchrony (IBS)—the alignment of neural activity between individuals—as an indicator of collaborative success. Using functional Near-Infrared Spectroscopy (fNIRS) hyperscanning, we investigated IBS in 30 dyads of undergraduate students engaged in a naturalistic three-phase Zoom seminar comprising lecture viewing, interactive discussion, and a presentation. Inter-brain synchrony between partners was computed in the prefrontal cortex, and outcomes were assessed via relational satisfaction questionnaires and standardized ratings of presentations. Results showed that IBS emerged predominantly during active discussions, but not during passive lecture viewing, underscoring the importance of interactive engagement in generating neural alignment. Crucially, higher IBS during discussion predicted both greater group relational satisfaction and improved task performance. These findings extend prior evidence that IBS supports cooperation, demonstrating that neural synchrony can occur even without physical co-presence and is associated with both performance and satisfaction in virtual educational settings.

## Introduction

Collaborative learning, grounded in socio-cultural theories of learning (Vygotsky, 1978) and often implemented in face-to-face settings since the 1970s, encourages students to engage in problem-solving and co-construction of knowledge through meaningful dialog and mutual support (Johnson and Johnson, 2009). In recent years, the COVID-19 pandemic and widespread adoption of platforms like Zoom have accelerated a shift toward online collaborative learning (Chen and Tan, 2024; Vahle et al., 2023). Undergraduate seminars traditionally characterized by face-to-face interactive activities have now transitioned significantly to virtual formats. As educational institutions continue to adopt remote learning platforms, examining how virtual settings affect group dynamics and cooperative task outcomes becomes increasingly relevant to optimizing educational strategies and student engagement.

Research has identified several key factors that influence the effectiveness of online collaborative learning, one of which is group dynamics and satisfaction (Yang et al., 2024). For instance, Ku et al. (2013) found that team dynamics and acquaintance among teammates were significantly associated with students’ satisfaction with online teamwork, accounting for over half the variance in teamwork satisfaction. Similarly, Bach and Thiel (2024) demonstrated that the quality of digital interactions, especially creating a sense of community, strongly influenced group climate, participation, and satisfaction. Cheng et al. (2023) added that perceived trust, interaction, and social support from peers and teachers were significant factors that help reduce cognitive load during online collaborative learning. These findings all highlight that relational quality, communication, and emotional safety are essential for effective online collaborative learning. Despite these rich insights, most existing studies rely on self-reported measures or observational data alone, which limit our understanding of the real-time cognitive and affective dynamics underpinning online collaboration (Kaliisa et al., 2025).

Recent advancements in neuroscience, particularly the use of hyperscanning techniques, have provided novel insights into group interactions through measures of inter-brain synchrony (IBS). IBS refers to the phenomenon where individuals’ neural activities become aligned, reflecting coordinated cognitive and attentional states during interpersonal interactions (Azhari et al., 2022; Kinreich et al., 2017). In studies on collaboration, IBS has been shown to emerge in regions associated with social cognition and executive functioning, such as the prefrontal cortex. For instance, Zhang et al. (2021) found that elevated synchrony was observed in the inferior frontal gyrus (IFG) when teams collaborated on decision-making involving reward incentives. Along the same vein, Zhou et al. (2022) demonstrated that pairs who engaged in a time estimation task showed greater synchrony in the dorsolateral prefrontal cortex (DLPFC) compared to controls. These studies highlight the relevance of IBS during interpersonal interactions in collaborative settings.

Previous research has demonstrated clear links between inter-brain synchrony and collaborative outcomes, specifically task performance and relational satisfaction among group members. Task performance, referring to the quality and effectiveness of task outcomes achieved by collaborative efforts, has consistently shown correlations with IBS levels. For instance, in a team-based problem-solving task, IBS, but not self-reported measures of group belonging, has been shown to be predictive of task performance (Reinero et al., 2021). IBS has also been observed during online cooperative gaming conducted without physical co-presence, suggesting that neural coupling can occur even in digital, non-face-to-face interactions (Wikström et al., 2022). Such findings underscore the potential for IBS to serve as an indicator of more cohesive cognitive processes among group members supporting effective collaboration in online contexts.

Group relational satisfaction, another critical variable, refers to the positive perceptions and satisfaction members experience regarding their group interactions and interpersonal relationships. While there is limited literature on IBS and group relational satisfaction, studies on IBS with social bonding in general have been conducted. In a recent study, Hinvest et al. (2025) examined dyads engaged in naturalistic conversations and demonstrated that those who reported a greater sense of shared identity with each other exhibited higher levels of IBS. Similarly, Algumaei et al. (2023) and Hoehl et al. (2021) suggested that IBS in socio-emotional brain areas support trust and the formation of social bonds, indicating relational satisfaction among group members.

However, research specifically examining inter-brain synchrony within online educational settings remains limited, with few studies having investigated the relationship between brain synchrony, task performance, and group relational satisfaction in naturalistic online pedagogical environments. Addressing this gap, the present study utilizes functional Near-Infrared Spectroscopy (fNIRS) to measure IBS among undergraduate students participating in a naturalistic online Zoom seminar—comprising a lecture, interactive discussion, and presentation components—can predict group relational satisfaction and task performance. This study is part of a larger ongoing project that examines the neural, physiological and behavioral components of online learning activities that support collaboration.

We embarked on this study with two central research questions: (1) Does inter-brain synchrony between pairs of undergraduates emerge in the lecture (passive co-viewing) and discussion (active interaction) conditions? (2) Does inter-brain synchrony between pairs of undergraduates predict group relational satisfaction and task performance in pairs of undergraduate students? For the first research question, we hypothesized that inter-brain synchrony will be observed during the discussion segment involving active interactions between participants, but not the lecture segment when participants were passively co-viewing a pre-recorded video together. Secondly, in line with previous research, we hypothesize that inter-brain synchrony will predict both group relational satisfaction and task performance.

## Methods

### Participants

A total of 30 dyads of undergraduate students were recruited via convenience sampling. Due to poor data quality, seven dyads were excluded, resulting in a final sample of 23 dyads (*N* = 46). Participants were aged between 21 and 30 years (*M* = 24.41; SD = 2.8). The gender composition consisted of 13 mixed-gender (male–female) pairs (*N* = 26), 7 male–male pairs (*N* = 14), and 3 female–female pairs (*N* = 6). Participants were enrolled across diverse academic disciplines, predominantly within the Social Sciences (56.5%) (e.g., Psychology, Social Work, Law), followed by Business and Management (28.3%) (e.g., Accounting, Marketing, Air Transport, Supply Chain), STEM fields (8.70%) (e.g., Engineering, Computer Science, Data Science), and Health Sciences (6.5%) (e.g., Dental Surgery, Diagnostic Radiography). During recruitment all participants were confirmed to have experience with online learning platforms such as Zoom during their coursework. Key pre-experimental precautions involved instructing participants to refrain from caffeine or vigorous physical activity for at least 2 h before the experiment to minimize physiological variability in fNIRS recordings. Participants were also assigned anonymous identifiers to ensure that prior familiarity between dyad members did not confound the results. This study was approved by the Institutional Review Board of the Singapore University of Social Sciences with protocol ID: APL-0184-2022-EXP-07.

There was an attrition rate of 23.3% for this group of participants, primarily due to noise detected by fNIRS signals. This is consistent with attrition rates derived from other fNIRS studies, where the loss of data poses a significant risk due to participants’ natural hair and atmospheric noise (Baek et al., 2023; Liu et al., 2024). Additionally, a study focusing on synchrony between dyads poses an additional constraint wherein the loss of one participant’s data affects the analysis of pair synchrony.

### Hyperscanning modality

fNIRS hyperscanning offers advantages over other hyperscanning modalities in providing ecologically valid yet spatially sensitive results. While fMRI and MEG may confer stronger spatial resolution, fNIRS enables participants to move, speak, and engage naturally without strict motion constraints, which is critical for studying real-time social communication (Czeszumski et al., 2020). Similarly, while EEG provides higher temporal resolution, fNIRS offers better spatial resolution for monitoring prefrontal activity and is less susceptible to motion and muscle artifacts that commonly occur in dialog-based tasks (Li et al., 2022). This makes the fNIRS hyperscanning modality ideal for simultaneously measuring cortical hemodynamic activity from two participants during naturalistic social interactions, whether face-to-face or online, as these contexts inherently involve spontaneous verbal exchanges, gestures, and other nonverbal cues (Zhao et al., 2024).

### Task procedure

All collaborative sessions were conducted remotely via Zoom, reflecting realistic hybrid learning and work settings. The participants were placed in separate rooms where each dyad completed a structured sequence comprising three main steps. During which, researchers were facilitating the session and monitoring the participants’ activity via the same Zoom call from another room.

First, in the (10 min) viewing phase, participants watched a recorded lecture about social media usage in teenagers that provided a shared context for the collaborative task. The recorded lecture included a neutral problem statement about excessive social media usage among teenagers, setting the stage for the subsequent sections of the task. Next, in the (20 min) discussion phase, the participants were randomly assigned to three different task conditions: brainstorming, problem solving, and cognitive conflict, where they then engaged in a discussion about the problem statement in a live collaborative dialog to solve the assigned problem.

The three task conditions were selected based on McGrath’s (1984) Group Task Circumplex, where tasks are split into four prototypical categories: generate, choose, negotiate and execute, where the cognitive demands and requirements for interdependent collaboration vary. Straus’s (1999) empirical validation of McGrath’s (1984) cooperation-conflict axis reinforces the circumplex’s relevance for structuring experimental designs on group interaction. By assigning triads to perform an idea-generation task, an intellective problem-solving task, and a judgment task, Straus observed systematic variations in agreement, disagreement, and process-oriented communication as interdependence and conflict increased. Building on these findings, the present study adapted three tasks spanning McGrath’s “generate–choose–negotiate” continuum. The brainstorming condition activity mimics that of the “generate” task type, reflecting the low-interdependence and high-divergence features of generative tasks. The problem-solving condition presented in the form of analytical problem-solving, required participants to coordinate efforts in finding acceptable solutions to the problem statement, capturing the consensus-driven features of choice-based tasks. The cognitive conflict condition, inspired by the “negotiate” task type, represented the high-interdependence, high-conflict demands of negotiation tasks, which are also associated with increased cognitive load and negative affect. This required participants to converge and reconcile opposing perspectives, thereby requiring increased coordination, discussion and cognitive effort. The “execute” task type was not utilised in this experiment as it does not correlate with the study’s conceptual scope of ideational collaboration (McGrath, 1984). This structured variation enabled us to examine how the affective climate of collaboration, ranging from positive to negative sentiment, relates to both relational satisfaction and performance outcomes, thereby extending McGrath’s theoretical framework and Straus’s empirical contributions into the affective domain.

**Figure 1:**
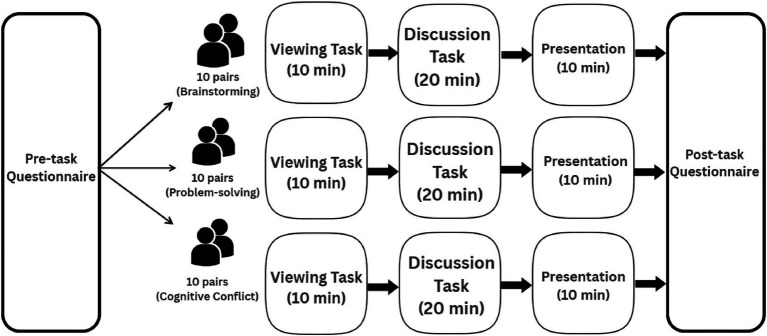
Experimental design illustrating the sequence of phases (viewing, discussion, presentation) and task-type allocation across participant dyads.

**Figure 2:**
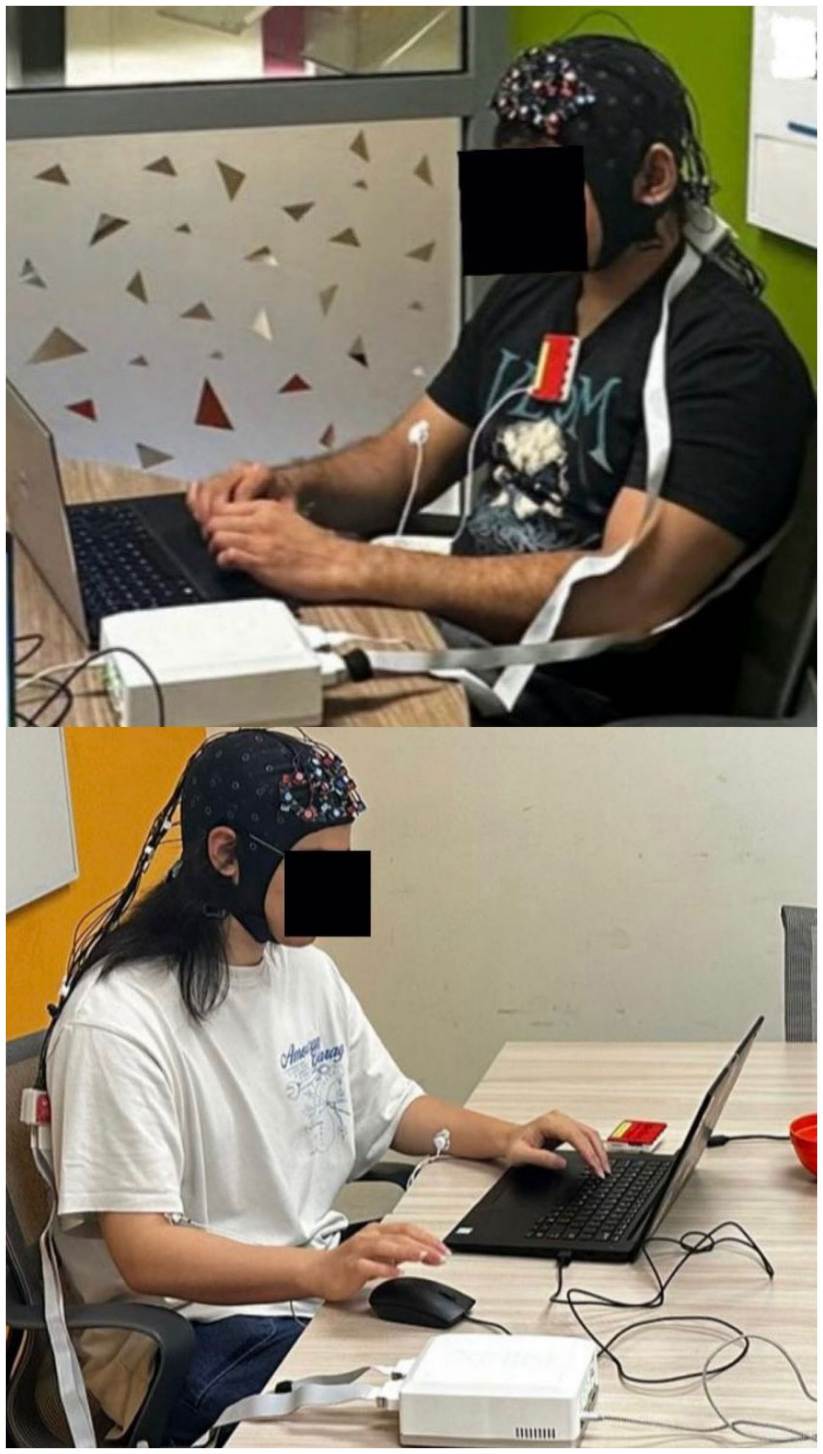
Experimental setup: participants seated in different rooms, connected via Zoom, each wearing an fNIRS device.

Finally, in the (10 min) presentation phase, participants delivered their joint output based on the condition-specific problem statement (see Figure 1). Participants were required to keep their video cameras turned on throughout the entire session. The experimental setup is illustrated in Figure 2, where each participant is seated in a separate room, connected over Zoom, and equipped with an fNIRS device. Following their presentations, each member of the pair was asked to complete a post-task questionnaire, which consists of the 12-item Relational Satisfaction questionnaire by Anderson et al. (2001).

### fNIRS hyperscanning setup

**Figure 3:**
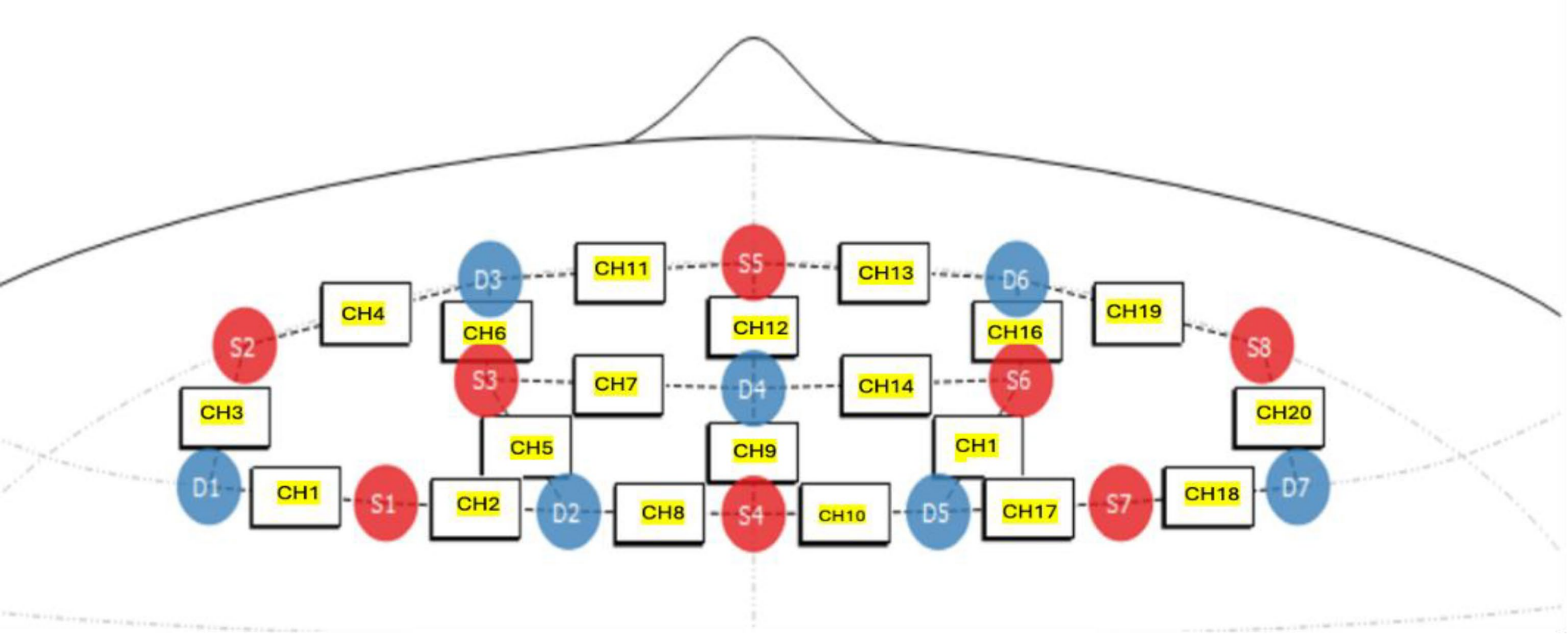
Montage of fNIRS optodes targeting the prefrontal cortex adapted from Aurora fNIRS software (version 2023.9.3-1).

The measurement of changes in oxygenated (HbO) and deoxygenated (HHb) hemoglobin in the prefrontal cortex was conducted using a NIRSport2 (NIRx Medical Technology LLC) continuous-wave fNIRS device. A multi-channel portable system (8 sources and 8 detectors, NIRSport 64 data channels) was used for data recording (see Figure 3). The customized prefrontal cortex source-detector arrangement was determined by the manufacturer. Eight sources and seven detectors were positioned in reference to Cz and the nasion, resulting in a total of 20 channels. The source optodes transmitted light at 760 and 850 nm, and data were sampled at a frequency of 10 Hz. Optode placement followed the international 10–20 system, targeting the prefrontal cortex. All fNIRS data were streamed wirelessly to a local computer using Aurora (version 1.4) software and saved in the Shared Near Infrared Spectroscopy Format (SNIRF; Tucker et al., 2022).

### Data preprocessing

Data preprocessing was conducted in Python (Version 3.13.1; Python Software Foundation, 2024) with the MNE-Python library (Gramfort et al., 2013). Coefficient of variation (CV) values were computed on raw input signals segmented by activity phase and source-detector channels. Raw signals were transformed into optical density (OD) using the standard logarithmic transformation and motion corrected with wavelet filtering, which was shown to outperform the default temporal derivative distribution repair method within MNE-Python (Iester et al., 2024). OD signals were decomposed using discrete wavelet transform with the Daubechies 5 wavelet to their maximum decomposition level permitted by signal lengths (*pywt*.*dwt_max_level*). Denoising was achieved by setting detail coefficients exceeding the threshold (as shown in the below equation) to zero with α=0.10 (Brigadoi et al., 2014; Molavi and Dumont, 2012).


$${\hat{w}}_{\mathrm{jk}}=\{\begin{array}{ll} w_{\mathrm{jk}} & \text{if}\;|w_{\mathrm{jk}}|\leq \tau ,\text{where}\;\tau =z_{1-\alpha /2}\cdot \frac{\text{median}(|w_{\mathrm{jk}}|)}{0.6745}\\ 0 & \text{otherwise} \end{array}$$

Where ${\hat{w}}_{\mathrm{jk}}$ and $w_{\mathrm{jk}}$ are the denoised and original detail coefficients at level j , with k elements

Reconstructed OD signals were passed through a zero-phase, band-pass, Kaiser-window-designed finite impulse response (FIR) filter (1,001st order, $F_{c}=[0.01,0.20]$ Hz, 60 dB attenuation) to isolate neurovascular signals relevant to cognitive processing (Pinti et al., 2019). This frequency range captures task-related hemodynamic activity while attenuating noise from sources such as respiration and cardiac pulsations. Additionally, filter order was dynamically reduced for signals shorter than 3,000 samples by constraining the maximum allowable filter order as shown below:


$$N_{\text{order}}={\left\lfloor \frac{L-1}{3}\right\rfloor }_{\mathrm{odd}}$$

Where L is signal length in samples.

Filtered OD signals were then transformed into HbO and HbR concentrations through the modified Beer–Lambert Law with partial pathlength factor of 6.0. Finally, inter-brain synchrony was computed as the maximum normalized cross-correlation achieved across all lags (≤ ± 5 s) between anatomically homologous channel pairs (i.e., channels occupying the same spatial locations across the two participants), calculated separately for each dyad and each task phase. Cross-correlations were used to conduct synchrony analyses, as it has been previously determined to be optimal for naturalistic neuroscience studies and found to generate more robust, reproducible results when compared to other computational approaches such as wavelet coherence (Bizzego et al., 2022).

Each recording was segmented into three distinct task phases: Viewing (VIEW), Discussion (DISC), and Presentation (PRES) using time markers extracted from a structured event log.

The maximum correlation value observed within a ± 5 s lag window was retained to reflect peak synchrony, accommodating potential inter-individual delays in the hemodynamic response. Given the exploratory scope and limited sample size of the present pilot study, peak cross-correlation values were then aggregated by averaging across all homologous channel pairs to obtain a global synchrony score for the whole prefrontal cortex (PFC) for each dyad and task phase. While the PFC is functionally heterogeneous, prior meta-analytic evidence demonstrates that cooperative tasks consistently elicit inter-brain synchrony within this region across diverse paradigms (Czeszumski et al., 2022), reinforcing its reliability as a central cortical hub for social interaction (Lim et al., 2024). Moreover, region-level averaging approaches have been adopted in previous fNIRS studies to derive stable connectivity indices under pilot or exploratory conditions (e.g., Ieong and Yuan, 2017), supporting the validity of a whole-PFC aggregation approach in the current study.

### Surrogate data

To correct for IBS simply resulting from simultaneous task engagement, baseline IBS values (by channel, wavelength, condition, and task phase) were computed from the average of all possible permutations of surrogate pairs and subtracted from the IBS values of real pairs (Sijben et al., 2025). The corrected IBS values were then used in all subsequent analyses.

### Dependent variables

Group Relational Satisfaction (GRS) was derived by averaging two individual ratings—one from each participant in a dyad. Task performance was assessed based on participants’ joint presentations. These were evaluated using a standardized rubric adapted from educational communication research (Peeters et al., 2010). Two independent raters graded each presentation on Slide Effectiveness, which included dimensions such as visual clarity, organization, critical thinking, and relevance of conclusions. Each dimension was rated on a 4-point Likert scale (1 = Needs Improvement to 4 = Excellent). The raters achieved an inter-rater reliability of Cohen’s Kappa value of 0.8113, indicating a strong understanding of the grading rubrics. Finally, peak inter-brain cross-correlation values during the discussion phase were extracted as the measure of neural synchrony.

### Statistical analysis

All formal statistical analyses were executed in R (Version 4.4.2; R Core Team, 2024). Channels with CV ≥ 7.5% were omitted from analyses. Gender pairings and wavelengths were included as covariates in each model.

## Results

### Descriptive statistics

**Figure 4:**
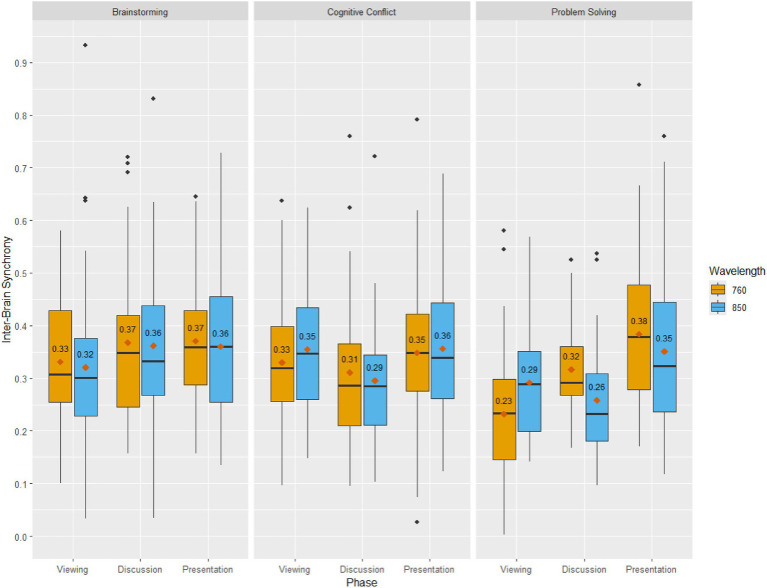
Descriptive boxplots of inter-brain synchrony by task, condition, and wavelength.

**Figure 5:**
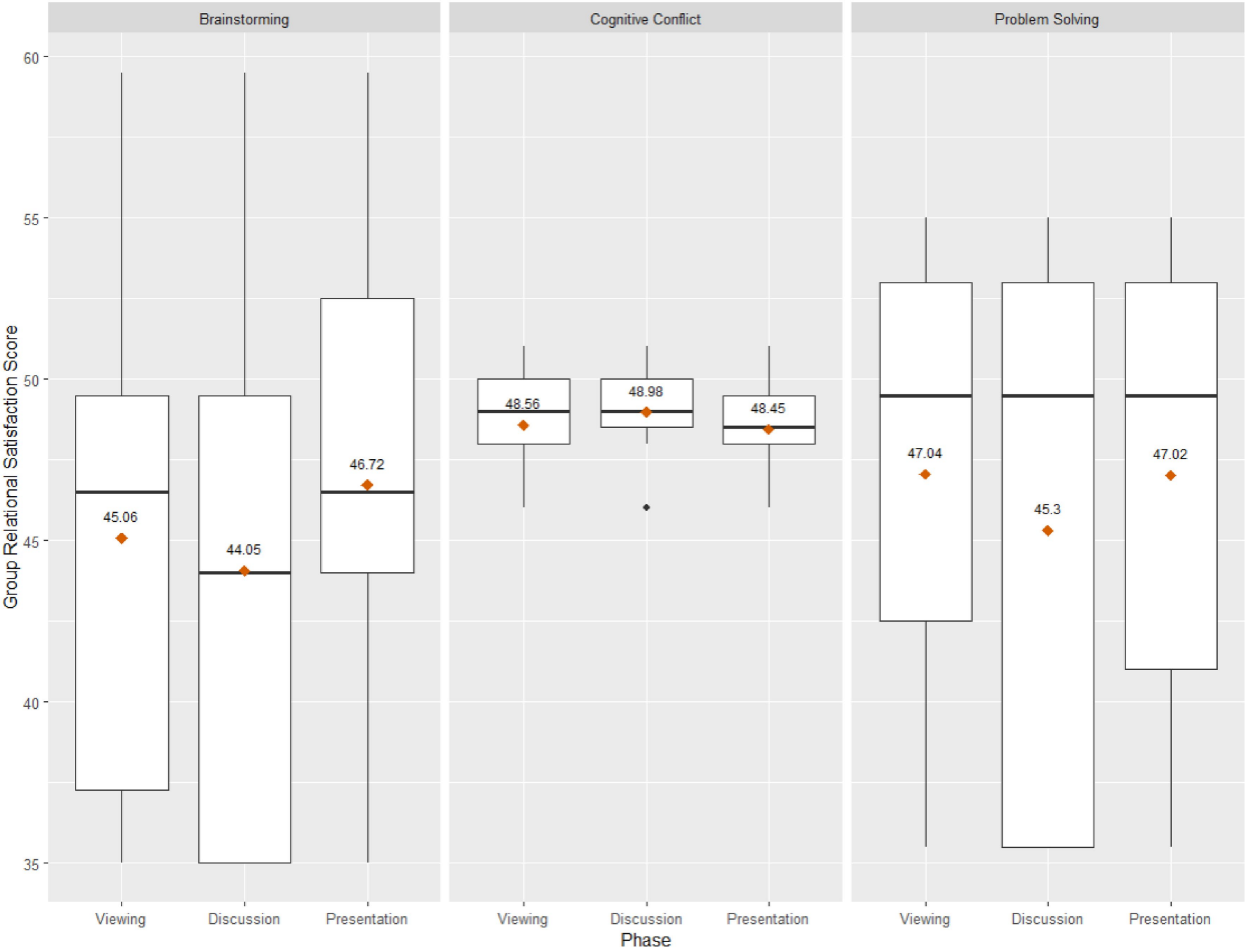
Descriptive boxplots of group relational satisfaction scores by task and condition.

Table 1 presents the descriptive statistics of Inter-Brain Synchrony (i.e., maximum correlation value) and Group Relational Satisfaction scores for the present sample. Figure 4 illustrates inter-brain synchrony by task, condition, and wavelength and Figure 5 depicts boxplots of group relational satisfaction scores by task and condition.

**Table 1:** Sample descriptive statistics.

	Brainstorming	Cognitive conflict	Problem-solving
Number of Dyads	6	8	9
Number of valid channels	323	369	277
Inter-brain synchrony	0.356 (0.129)	0.368 (0.116)	0.348 (0.125)
Group relational satisfaction score	45.463 (7.604)	48.594 (1.478)	46.718 (7.293)

### Inferential results

#### Effects of (within-subjects) phase and (between-subjects) task on IBS

A factorial ANOVA was conducted to examine the interaction effects between within-subjects phase (viewing, discussion, presentation) and between-subjects task (cognitive conflict, brainstorming, problem-solving) on IBS. There was a significant main effect of within-subjects phase, *F*(2, 957) = 13.387, *p* < 0.001, $\eta_{p}^{2}$ = 0.027; where pairwise comparisons of estimated marginal means revealed that IBS was significantly higher during presentation phase (*M* = 0.364, *SE* = 0.007) compared to both viewing (*M* = 0.313, *SE* = 0.007, *p* < 0.001) and discussion (*M* = 0.320, *SE* = 0.009, *p* < 0.001) phases. There was a significant main effect of between-subjects task, *F*(2, 957) = 7.157, *p* < 0.001, $\eta_{p}^{2}$ = 0.015; where pairwise comparisons of estimated marginal means revealed that IBS was significantly higher in brainstorming (*M* = 0.357, *SE* = 0.009) as compared to problem-solving (*M* = 0.307, *SE* = 0.009, *p* < 0.001).

**Figure 6:**
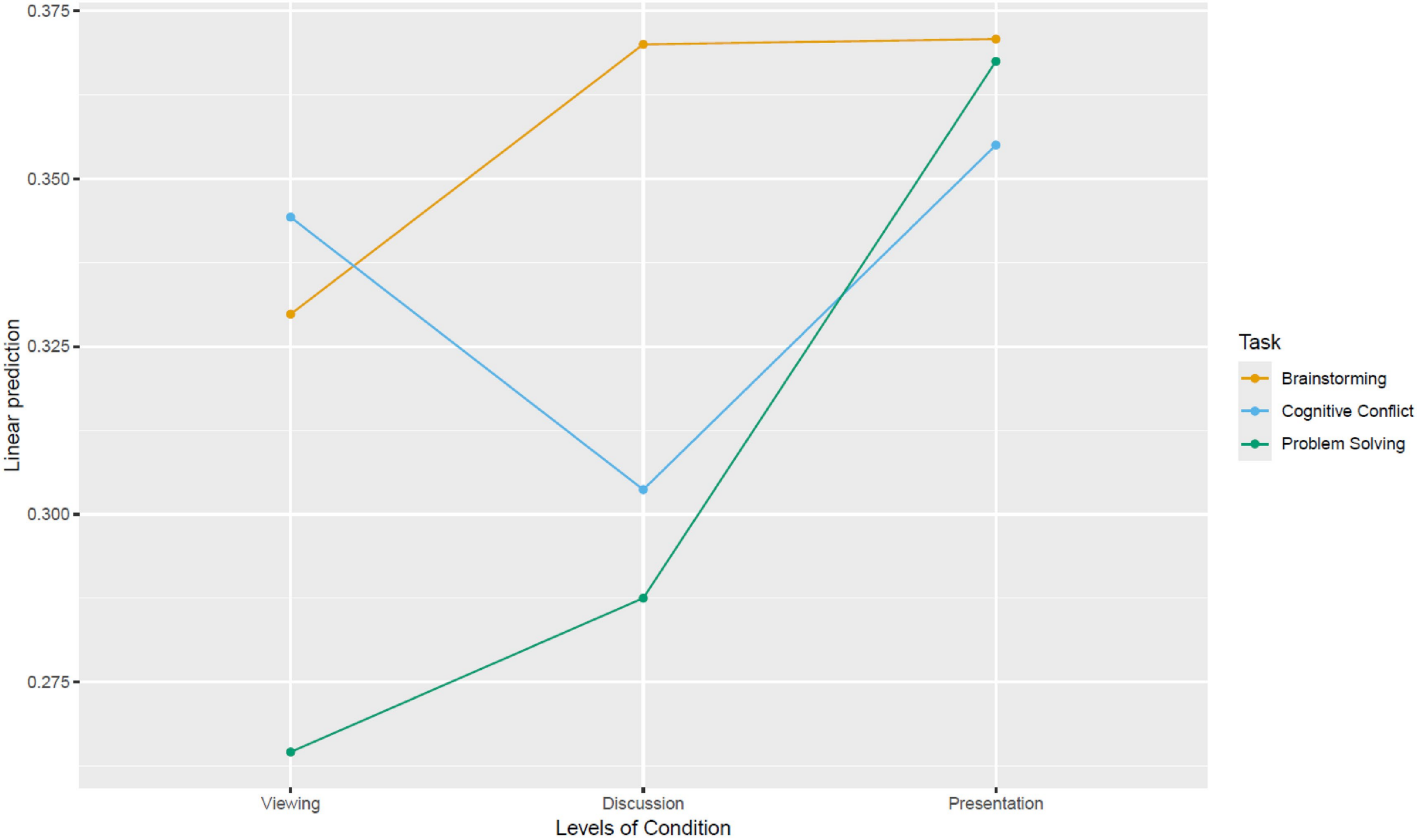
Interaction between phase and task on IBS.

There was also a significant interaction (see Figure 6) between phase and task on IBS, *F*(4, 957) = 6.114, *p* < 0.001, $\eta_{p}^{2}$ = 0.025. *Post-hoc* examination of simple effects highlighted that: (a) For brainstorming, IBS during presentation was only significantly higher than viewing (*Δ* = 0.041, *p* = 0.032); (b) for cognitive conflict, IBS during presentation was only significantly higher than discussion (*Δ* = 0.051, *p* = 0.019); and (c) for problem-solving, IBS during presentation was significantly higher than both viewing (*Δ* = 0.103, *p* < 0.001) and discussion (*Δ* = 0.080, *p* < 0.001).

**Figure 7:**
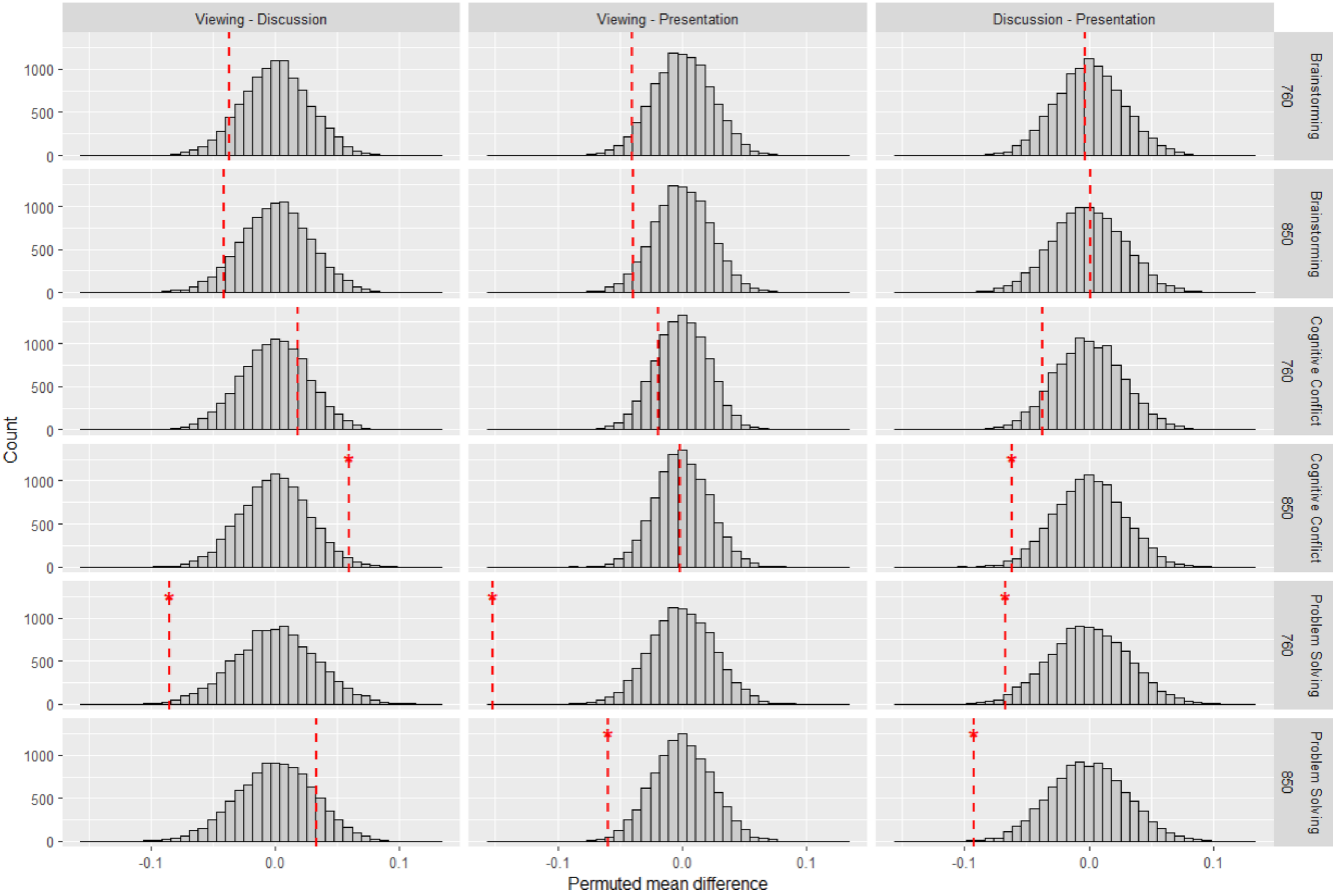
Permuted pairwise contrasts of IBS between phases. Red dashed lines indicate observed values. *p < 0.05.

**Figure 8:**
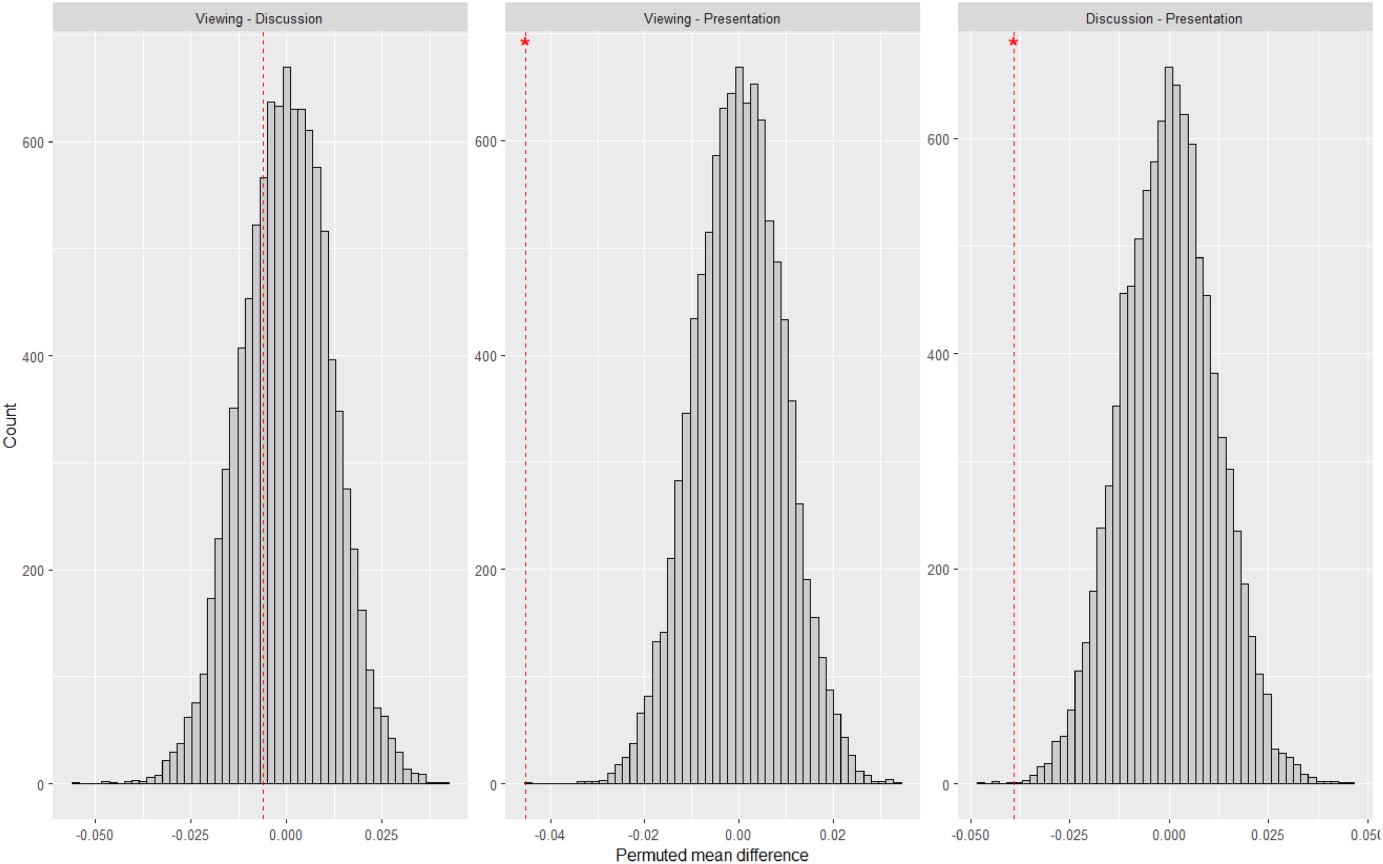
Permuted pairwise contrasts of IBS between phases across tasks and wavelengths. Red dashed lines indicate observed values. *p < 0.05.

Assumptions of normality of residuals were not met as assessed by Shapiro–Wilk test, *p* < 0.001 while homoscedasticity was met as assessed by Breusch–Pagan test, *χ*^2^(11) = 18.737, *p* = 0.066. Further constrained by our limited sample size, we attempted a supplementary non-parametric permutation test (np = 10,000) to corroborate our findings. Permutation tests are more flexible non-parametric tests that allow for comparisons of means (Berry et al., 2011; Holt and Sullivan, 2023). Results (see Figure 7) show that the main effects of phase (*p* < 0.001) and task (*p* < 0.001); and interaction effect between phase and task (*p* < 0.001) on IBS remain significant. Permuted pairwise contrasts stratified by task and wavelength are presented in Figure 8.

#### Effects of IBS during viewing on GRS

Next, results of a multiple linear regression model, *F*(8, 382) = 15.094, *p* < 0.001, $R_{\mathrm{adj}}^{2}$ = 0.224, failed to show any significant relationship between IBS during the viewing phase and GRS, *β =* 0.025, *SE* = 3.611, *p* = 0.737. However, male–female pairings reported significantly higher and lower GRS than male–male (*β = −*0.256, *SE* = 0.780, *p* < 0.001) and female–female (*β =* 0.267, *SE* = 0.778, *p* < 0.001) pairings, respectively. Dyads under the cognitive conflict task also reported significantly higher GRS than both those in the brainstorming task, *B =* 3.958, *SE* = 0.661, *p* < 0.001, and those in the problem-solving task, *β =* 2.029, *SE* = 0.795, *p* = 0.030.

#### Effects of IBS during discussion on GRS

Additionally, we estimated a multiple linear regression model, *F*(8, 180) = 9.039, *p* < 0.001, $R_{\mathrm{adj}}^{2}$ = 0.255, to examine whether IBS and task predicted GRS scores during the discussion phase. Results uncovered that: (a) IBS was significantly negatively correlated with GRS, *β =* −0.226, *SE* = 3.730, *p* = 0.015; (b) female–female (*β =* 0.199, *SE* = 1.168, *p* = 0.005) pairings reported significantly higher GRS scores than male–female pairings; and (c) there was a significant interaction effect between IBS and cognitive conflict task on GRS, *β =* 0.376, *SE* = 5.850, *p* = 0.029.

**Figure 9:**
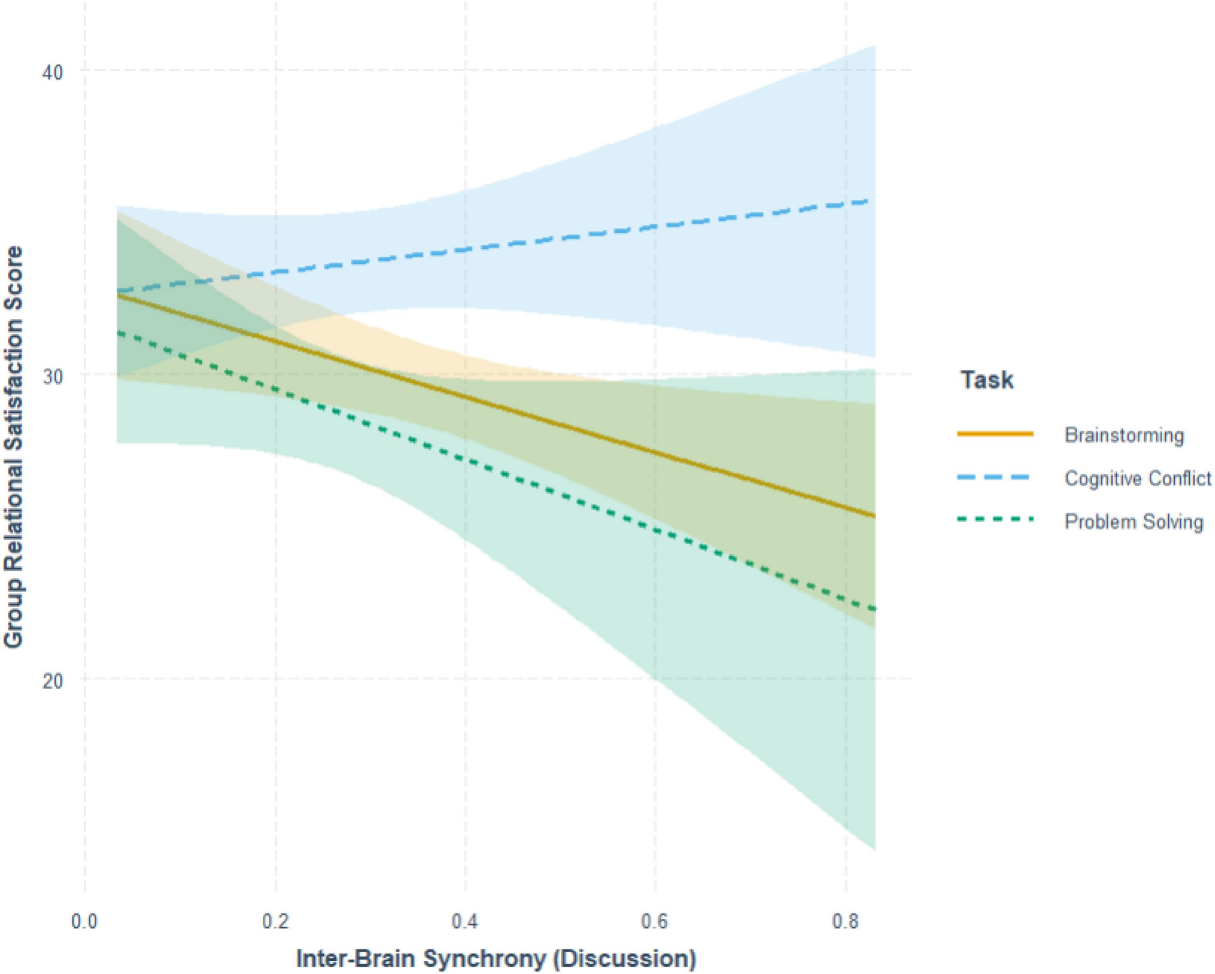
Interaction between IBS and (between-subjects) task on GRS.

Simple slopes analysis (see Figure 9) show that IBS was (a) significantly negatively correlated with GRS under the brainstorming task, *B* = −9.115, *SE* = 3.730, *p* = 0.015; (b) non-significantly correlated with GRS under the problem-solving task, *B* = −11.497, *SE* = 6.905, *p* = 0.098; and (c) non-significantly correlated with GRS under the cognitive conflict task, *B* = 3.745, *SE* = 4.542, *p* = 0.411. However, subsequent pairwise contrasts show that all slopes were not significantly different from each other.

#### Effects of IBS during viewing on presentation score

Furthermore, we estimated a multiple linear regression model, *F*(8, 368) = 50.423, *p* < 0.001, $R_{\mathrm{adj}}^{2}$ = 0.513, to examine whether IBS during the viewing phase and task predicted presentation scores. Results show that: (a) higher IBS was associated with higher presentation scores, *β =* 0.141, *SE* = 1.592, *p* = 0.017; (b) participants under the problem-solving task scored significantly higher than those in the brainstorming task, *β =* 0.418, *SE* = 0.867, *p* < 0.001; and (c) both male–male, *β =* 0.454, *SE* = 0.373, *p* < 0.001, and female–female, *β =* 0.076, *SE* = 0.344, *p* = 0.047 pairings achieved significantly higher scores than male–female pairings.

#### Effects of IBS during discussion on presentation score

Likewise, we estimated a multiple linear regression model, *F*(8, 172) = 35.529, *p* < 0.001, $R_{\mathrm{adj}}^{2}$ = 0.606, to examine whether IBS during the discussion phase and task predicted presentation scores. Results show that: (a) there was no significant effect of IBS during discussion on presentation scores *β <* 0.001, *SE* = 1.552, *p* = 0.992; (b) participants under the problem-solving task scored significantly better than those in the brainstorming task, *β =* 0.502, *SE* = 1.069, *p* < 0.001; (c) participants under the cognitive conflict task scored significantly worse than those in the brain storming task, *β = −*0.281, *SE* = 0.882, *p* = 0.032; and (d) male–male pairs scored significantly better than male–female pairs, *β =* 0.512, *SE* = 0.455, *p* < 0.001.

#### True versus surrogate pairs

Lastly, to examine if any synchronicity observed merely resulted from simultaneous task performance, surrogate data was generated through arbitrarily random pairings of independent participants. Results of a two-tailed, two-sample *t*-test show that IBS between true pairs were significantly different from IBS between surrogate pairs, *t*(1267) = −22.945, *p* < 0.001. This suggests that IBS emerged due to specific interactions between pairs of participants, and not simply a result of similar cognitive processing when participants engaged in the same tasks.

## Discussion

The objectives of this study were to investigate the occurrence of inter-brain synchrony (IBS) between undergraduate students participating in a naturalistic online Zoom seminar and to examine whether this synchrony predicts group relational satisfaction and task performance. Specifically, we aimed to determine if IBS emerges during the passive co-viewing of a pre-recorded lecture and during active interactive discussion and presentation. Furthermore, we explored whether such synchrony would predict relational satisfaction among group members and their task performance. Our hypotheses were twofold: first, that brain synchrony would occur only during active discussions and not during passive viewing, and second, that synchrony observed during the discussion would predict both relational satisfaction and task performance.

### Phase-related dynamics of IBS

Our findings partially supported these hypotheses. Contrary to our initial expectation, significant IBS was observed primarily during the presentation phase rather than the discussion phase, as revealed by the factorial ANOVA results. Pairwise comparisons indicated that IBS during the presentation phase was significantly higher than both viewing and discussion phases. This pattern suggests that the emergence of synchrony was strongest when dyads jointly presented their collaborative output, a phase characterized by high levels of coordination, turn-taking, and shared attention. The observation of significant inter-brain synchrony during the joint presentation segment across all tasks aligns with previous research emphasizing the role of active, goal-directed, interpersonal interactions in facilitating alignment of brain activities. In a meta-analysis of cooperation studies, Czeszumski et al. (2022) revealed that all 13 studies reported significant IBS, particularly in the prefrontal cortex region, across diverse types of active cooperative tasks. The presentation phase required participants to synchronize not only speech and timing but also shared executive control, mutual prediction, and real-time monitoring of each other’s verbal and nonverbal cues, processes previously linked to prefrontal coupling (Reinero et al., 2021).

### Task-specific differences

Beyond phase-related differences, *post-hoc* analyses revealed distinct synchrony patterns across the three collaborative tasks. A significant increase in IBS was observed during Brainstorming compared to Problem Solving, with a similar but descriptive upward trend relative to Cognitive Conflict. The free-flowing exchange of brainstorming may transiently heighten alignment through shared attention and perspective-taking. This is consistent with unstructured, co-creative dialog that encourages spontaneous idea of exchange and mutual responsiveness (Straus, 1999). Participants are free to build on one another’s ideas in an open and collaborative manner. This fluid exchange likely promotes continuous mutual attention, spontaneous turn-taking, and verbal mirroring, processes that enhance neural coupling. In contrast, cognitive conflict and problem-solving tasks, which demand more structured, goal-oriented, and evaluative forms of interaction (Straus, 1999), exhibited lower synchrony during discussion but a sharper rise during the presentation phase. This reflects a delayed alignment that emerges when consensus must be enacted rather than negotiated. The structured and evaluative nature of cognitive conflict may transiently disrupt coupling as partners oscillate between agreement and disagreement while reconciling differing viewpoints (Hirsch et al., 2021). Similarly, our problem-solving activity emphasized evaluating options and reasoning independently fosters parallel rather than co-constructed processing. Such goal-directed divergence reduces the shared attentional focus that undergirds neural coupling (Fishburn et al., 2018).

Additionally, Ma and Liu (2024) similarly highlighted that synchrony arises from shared attentional and cognitive alignment during socially engaging or goal-directed activities. This supports our finding that IBS occurred during a joint presentation segment between the dyads. Taken together, these findings position IBS as a flexible neural mechanism underlying joint cognitive regulation, a dynamic process through which partners calibrate shared attention, prediction, and control in response to situational demands. The data support recent arguments that synchrony is modulated less by relational closeness and more by situational engagement and communicative reciprocity (Ma and Liu, 2024). The concurrent rise in IBS across all task types during joint presentation underscores that synchrony may represent the neural instantiation of shared intentionality—the alignment of mental states needed to accomplish collective goals. Our finding also aligns with Wikström et al. (2022), who demonstrated that neural synchronization can occur without physical co-presence during cooperative online activities. This supports our finding that remote interactions in virtual educational contexts can foster meaningful neural coupling. The lack of observed synchrony during passive co-viewing reinforces the critical role of active engagement and shared cognitive and attentional processes in achieving neural synchronization.

### Performance-related findings

Beyond its role as a neural indicator of shared intentionality, IBS also predicted meaningful behavioral outcomes. The positive correlation we found between brain synchrony and task performance suggests that cognitive alignment during collaborative discussions facilitates more efficient information processing, collective decision-making, and enhanced problem-solving efficacy (Reinero et al., 2021). Indeed, elevated synchrony observed in brain regions associated with executive functioning and social cognition has been consistently linked to improved cooperative outcomes, such as better decision-making and problem-solving capabilities (Zhang et al., 2021; Zhou et al., 2022). In addition to task performance, the observed relationship between brain synchrony and group relational satisfaction reinforces previous findings on the social and affective dimensions of IBS. Previous studies have shown that synchronized brain activity correlates with enhanced interpersonal rapport, social connectedness, and cooperative behavior, which contributes to overall relational satisfaction within groups (Algumaei et al., 2023; Hinvest et al., 2025; Hoehl et al., 2021). Collectively, these results suggest that IBS reflects not only neural coordination but also functional markers of both cognitive efficiency and relational harmony. Extending this to virtual learning contexts, our findings highlight that fostering conditions that promote neural synchrony (i.e., such as structured collaboration, open dialog, and shared attentional focus) may optimize both interpersonal dynamics and performance outcomes. Our results extend these findings to the naturalistic context of online undergraduate seminar settings, suggesting that IBS between students during online seminars can serve as an indicator of task performance and interpersonal satisfaction, even in the absence of physical co-presence. Thus, fostering interactions that enhance IBS may be crucial in optimizing relational dynamics and cooperative success in virtual learning environments.

### Limitations

Despite the promising insights, this study has several limitations that should be addressed in future research. Firstly, the relatively small sample size and specific context of a Zoom-based undergraduate seminar limit the generalizability of our findings. Future studies should include larger and more diverse samples across different educational contexts to validate these results. Thirdly, we only assessed brain synchrony during a single session. Future longitudinal studies could offer a deeper understanding of how neural synchronization evolves over time in relation to sustained collaborative and relational outcomes. Finally, our research was limited to examining synchrony in the prefrontal cortex (PFC) due to the methodological constraints of fNIRS. Expanding the measurement to include other brain regions, particularly those implicated in social cognition and emotional processing, could provide a more comprehensive understanding of the neural underpinnings of collaboration and relational satisfaction.

## Conclusion

This study demonstrates that inter-brain synchrony occurs specifically during active interactive discussions in online undergraduate seminar contexts. We demonstrated that higher synchrony was associated with enhanced group relational satisfaction and improved task performance, underscoring the importance of fostering active interactions in remote learning environments. These findings highlight the viability of hyperscanning techniques, particularly fNIRS, for exploring the neural basis of effective collaboration and relational dynamics in educational settings.

## Data Availability

The raw data supporting the conclusions of this article will be made available by the authors, without undue reservation.
